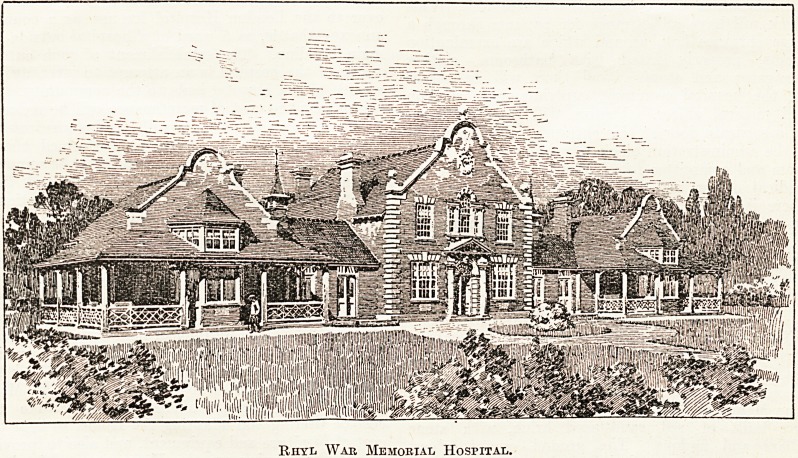# The Prince in His Principality

**Published:** 1923-12

**Authors:** 


### THE PRINCE IN HIS PRINCIPALITY.

### A HOSPITAL TOUR.

DURING his recent visit to the Principality the Prince of Wales laid the foundation-stone of the new wing of the Carnarvonshire and Anglesey Infirmary at Bangor, the foundation stone of the War Memorial Hospital at Wrexham, and opened the Rhyl War Memorial Hospital.

The new wing of the Bangor Infirmary, which is to cost nearly £16,000, consists of a two storey block containing general wards for fifty patients, private and children's wards, with verandahs on both sides and front to take advantage of the sun at all times of the day. A fire-escape stair is provided at the south corner of the ward block, and a covered way connects the first floor of the new blocks with the existing building. Under this covered way is the out-patients' entrance. The present children's ward is to be replaced by a two storey building giving accommodation for isolation wards. At the east end of the existing building a new block of buildings three storeys in height will be erected containing nurses' and staff common rooms on the ground floor and their bedrooms on the two floors above, with direct communication with the present building, the warming apparatus being arranged in the basement. The present hospital will be entirely remodelled, special attention being paid to the operating theatre and the kitchen department. The former has been considerably enlarged and rooms have been provided for anæsthetics, recovery wards, changing-rooms and sterilising-room.

### The Prince in the Wards.

The general appearance of the new building has been made to harmonise as nearly as possible with the existing range. Internally, the walls will be plastered and tiled, all angles being rounded to minimise the collection of dust and dirt, and the floors will be covered with a patent preparation which will have the appearance of wood block. The construction of the new work throughout will be fire-proof, the floors being built up of hollow concrete blocks. At the conclusion of the ceremony His Royal Highness expressed a wish to see the wards, and was conducted by Mrs. Maden (the matron) and Mr. Huw R. Gruffydd (the appeal secretary), who was formerly Assistant Secretary at the Royal Northern Hospital and subsequently Secretary of the County and County Borough Infirmary, Londonderry.

Rhyl War Memorial Hospital.

### Wrexham War Memorial Hospital.

This hospital is the result of two converging movements. On the one hand there was the desire to provide a worthy memorial to the men of East Denbighshire, and on the other there was the pressing need, growing stronger from year to year, of more adequate hospital accommodation. The existing infirmary situated in rather a noisy centre, is ninety years of age this year. It was built to meet the requirements of Wrexham when it was a small country town. To-day, Wrexham is the immediate centre of a population of 100,000, which is constantly increasing, so that the provision of more hospital accommodation has become an urgent and pressing necessity. The new hospital is intended to meet fully these two aspirations. In design, appliances and equipment no effort has been spared to bring it into correspondence with the latest developments of medical science. In addition to the site, the mansion opposite, and the two houses on the right, which are a free gift from the late Mr. John Jones, the estimated cost of the new hospital is £70,000.

The greater part of that amount has already been received. The balance has nearly all been promised, so that there is every prospect of the hospital being opened a year hence absolutely free of debt. In laying the foundation stone His Royal Highness said that he looked upon it as a great privilege to be asked to perform the ceremony, and congratulated those present most heartily on the wisdom of choosing as a memorial of the hundreds of gallant men from Wrexham who lost their lives in the Great War, this hospital, which will prove of great benefit and service to this town and district.

### The New Building.

The new hospital is planned on the principle of self-contained units conveniently and economically arranged from an axial corridor leading east and west through the old house. The admission and administration block forms the centre unit. This block will have an entrance for patients through a large entrance hall, around which are examination rooms, properly fitted with bathroom, clothes fumigator and storage, and there will be offices for the hospital secretary, superintendent, matron and medical officer. Visitors will gain admission by a separate door at this entrance. The first floor of the administration block is occupied by a board room and committee room, a suite of rooms for the medical officer, and another suite for the matron. There is a self-contained surgical unit, comprising up-to-date operating theatres, with requisite adjuncts, all on the first floor level. The ground floor of the unit provides the eye wards. The buildings are being constructed with red brick facings, stone dressings and slated roofs.

### Rhyl Memorial Hospital.

The Prince of Wales opened the Memorial Hospital at Rhyl, which has been erected by the town at a cost of £13,000, and by his permission it is to be called the Prince Edward War Memorial Hospital. It is a handsome structure built of red sand-faced brick, with stone pillars and facings. The ground covers about 1¼ acres, and the building is set well back from the road. It consists of a men's ward (6 beds), women's ward (6 beds), 4 private wards, matron's and nurses' sitting-rooms—all on the ground floor. At the rear of these rooms are a thoroughly up-to-date operating theatre with anæsthetic room adjoining, an X-Ray room with dark-room attached, a dispensary, consulting-room, baths, lavatories, kitchens and scullery with the necessary adjuncts. The centre block is two storeys high, having 5 bedrooms for the staff, bath, linen rooms and store rooms. The whole is warmed by a hot-water system, with a separate steam-heating apparatus for steam cooking, sterilizing, etc., and a separate domestic hot-water service. The building is lighted by gas and electricity in all rooms. The hall, octagonal in shape, is faced with white marble slabs, bordered by dark-green marble with black marble slips. On one of the white marble slabs the names of the Rhyl men who lost their lives in the Great War are enrolled. The floors of all the corridors are composed of terazzo, those of the wards and private rooms being of teradura. A handsome polished teak staircase gives access to the rooms above.

## Figures and Tables

**Figure f1:**